# Seasonal variation in the ultrasonic vocal activity of Humboldt's flying squirrel (*Glaucomys oregonensis*)

**DOI:** 10.1002/ece3.70344

**Published:** 2024-09-23

**Authors:** Travis A. Farwell, Barbara Clucas

**Affiliations:** ^1^ Department of Wildlife Cal Poly Humboldt Arcata California USA

**Keywords:** activity patterns, communication, function, nocturnal, small mammals, ultrasonic vocalizations

## Abstract

Most mammals rely on vocal communication to increase survival and reproductive success. While the functions of audible vocalizations have been well‐studied across mammal species, ultrasonic vocalizations in small mammals outside of bats are less understood. North American flying squirrel species (*Glaucomys* spp.), including the newly described Humboldt's flying squirrel (*Glaucomys oregonensis*), produce numerous call‐types that extend into the ultrasonic range. To investigate the potential function of ultrasonic call‐types in the Humboldt's flying squirrels, we used ultrasonic recorders to record squirrels in the wild across multiple seasons to determine if there are temporal and seasonal patterns in nightly vocal activity and rates of different call‐types. We recorded Humboldt's flying squirrels in two geographic locations ‐ Humboldt and San Bernardino counties—in California from 2018 to 2022 in the summer and winter across multiple study areas. We found that although seasonal weather conditions differ between locations, flying squirrels in Humboldt and San Bernardino had similar vocal activity patterns across nightly active periods between locations and between summer and winter. Nightly activity patterns of when the three main chirp‐like call‐types (arc chirps, tonal chirps, upsweeps) were given varied between seasons in both geographic locations, and these call‐types were given at greater rates in the summer in San Bernardino, but rates did not vary by season in Humboldt. Trills, the most structurally complex of the four main call‐types, were produced more in the summer than in winter, and also differed in their nightly activity patterns, in both geographic locations. Flying squirrels may use certain call‐types earlier or later in the nightly active period due to their potentially varying functions, and may produce more trills in the summer coinciding with the breeding season. Further understanding of the function of different call‐types can provide insight into social, foraging, and antipredator behavior of this nocturnal and elusive species.

## Graphical abstract

North American flying squirrel species, including the newly described Humboldt's flying squirrel (*Glaucomys oregonensis*), produce numerous call‐types that extend into the ultrasonic range. To investigate the potential function of ultrasonic call‐types in the Humboldt's flying squirrels, we used ultrasonic recorders to record squirrels in the wild across multiple seasons to determine if there are temporal and seasonal patterns in nightly vocal activity and rates of different call‐types. We found nightly and seasonal variation in call‐types, most notably that the most acoustically complex call type (trill) was given more often in the summer than winter suggesting a potential mating or territorial function.

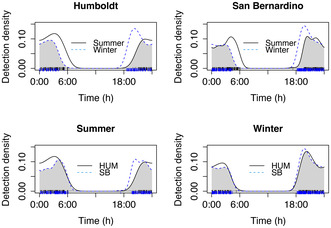

## 1 INTRODUCTION

Acoustic communication plays an integral role in the life history of most terrestrial mammals, with almost every species using some form of vocalization (with the exception of certain marsupial and heteromyid species; Chen & Wiens, 2020). In general, the frequencies of vocalizations produced by terrestrial mammals are related in part to their body size (Fletcher, 2004; Martin et al., 2017), with smaller mammals being more capable of producing relatively higher frequency vocalizations including ultrasonic vocalizations (USVs; i.e., above 20 kHz). Certain small mammal species are even able to produce both ultrasonic and audible vocalizations by using different laryngeal mechanisms (reviewed in Boulanger‐Bertolus & Mouly, 2021). In addition to body size, nocturnality and gliding behavior have been shown in mammals to be positively related to increased minimum vocal frequencies and increased maximum vocal frequencies, respectively (Newar et al., 2024). Terrestrial mammalian USVs were first described in bats (Pierce & Griffin, 1938), but were later found in a wider variety of species including rodents (e.g., Schleidt, 1948), insectivores (e.g., Gould et al., 1964), and some small primates (e.g., Pola & Snowdon, 1975). In rodents, USVs have been described in approximately 50 or more of the currently estimated 2923 species (Burgin et al., 2018; Sales, 2010), and it is possible that the use of USVs is present in additional species that have yet to be tested (Newar & Bowman, 2020).

USVs are well known in bat echolocation; however, there are only a few rodent species in which echolocation has been demonstrated (e.g., soft‐furred tree mice, *Typhlomys* spp.; He et al., 2021; Panyutina et al., 2017), and most behavioral studies on rodents have found that USVs serve other functions. In general, known functions of rodent USVs include aggressive displays (Kapusta et al., 2007), territorial defense (Rieger & Marler, 2018), courtship behavior (Hoffmann et al., 2012; Pultorak et al., 2017), kin recognition (Blake, 2012; Mandelli & Sales, 2004; Rabon Jr et al., 2001), and predatory alarm calls (Wilson & Hare, 2004, 2006). The current understanding of rodent USV function is mainly derived from studies focused on Muridae (e.g., rats and mice; Kalcounis‐Rueppell et al., 2010; Takahashi et al., 2010) and Cricetidae (e.g., voles; Kapusta et al., 2007), with relatively fewer studies focused on Sciuridae (e.g., ground squirrels and flying squirrels; Eiler & Banack, 2004; Gilley et al., 2019; Wilson & Hare, 2006).

The understanding of potential behavior functions of USVs in Sciuridae is limited. Several species of ground squirrels are known to produce calls extending into the ultrasonic range and have been shown to potentially serve antipredator functions (Eiler & Banack, 2004; Wilson & Hare, 2006). Richardson's ground squirrels (*Urocitellus richardsonii*) produce and react to USVs among conspecifics for predator avoidance and vigilance (Wilson & Hare, 2004, 2006), and golden‐mantled ground squirrels (*Callospermophilus lateralis* and *C. saturatus*) were discovered to use USVs for predator‐specific alarm calls (Eiler & Banack, 2004). North American flying squirrel species (*Glaucomys* spp.) are also known to produce USVs (Farwell et al., 2024; Giacalone‐Madden, 1976; Gilley, 2013; Shook, 1976), but their functions have not been systematically studied (Diggins, 2021; Newar & Bowman, 2020). While echolocation was a hypothesized function for USVs in flying squirrels due to their arboreal, gliding, nocturnal behavior, evidence has not supported this premise (Chattin, 1969), and anecdotal evidence suggests a communicative function. Across the three *Glaucomys* species, 29 distinct high‐frequency call‐types have been described: 10 produced by the northern flying squirrel (*G. sabrinus*), 27 by the southern flying squirrel (*G. volans*), and at least 7 by the Humboldt's flying squirrel (*G. oregonensis*) (Farwell et al., 2024; Gilley et al., 2019). The relatively large and diverse ultrasonic vocal repertoire of flying squirrels in North America further suggests the potential communicative significance of these vocalizations (Blumstein & Armitage, 1997). In addition, the potentially larger call repertoire in the southern flying squirrels may be related to the greater social behavior they exhibit compared to the northern flying squirrels and Humboldt's flying squirrels, as measured by their propensity to nest communally (Olson et al., 2018). All three North American flying squirrel species have been documented nesting communally (Carey et al., 1997; Cotton & Parker, 2000) and southern flying squirrels are known to exhibit this behavior throughout the year and in areas (e.g., Florida) where winter temperatures are mild (Garroway et al., 2013; Layne & Raymond, 1994). Communal nesting behavior thus might confer social benefits in addition to thermoregulation, such as increased mating and foraging opportunities and reduced predation risk (Garroway et al., 2013; Layne & Raymond, 1994; Olson et al., 2018), and vocal communication can be used to facilitate and coordinate such behaviors.

Typical approaches to understanding the function of vocalizations in a species would involve investigating the contexts in which different call‐types are given and how conspecifics respond; however, due to their elusive, nocturnal behavior patterns, observing and recording flying squirrels simultaneously in the wild is challenging. Nevertheless, flying squirrel vocalizations can be recorded with ultrasonic recorders in the wild, and the temporal and seasonal activity rates of different call‐types can be examined for patterns that could provide insight into their functions, similar to what has been done with birds and cetaceans (Au et al., 2000; Gil & Llusia, 2020; Pérez‐Granados & Schuchmann, 2023; Redaelli et al., 2022). As a first step in investigating the potential function of call‐types in the Humboldt's flying squirrels, we recorded squirrels in the wild across multiple seasons to determine if there are temporal and seasonal patterns in rates of different call‐types.

The Humboldt's flying squirrel is a recently described cryptic species, previously thought to be the same species as the northern flying squirrel (Arbogast et al., 2017). The Humboldt's flying squirrel geographic range is yet to be fully resolved, but the northern limit is likely southern British Columbia in Canada extending southward through Washington and Oregon in the United States and down through California, including an isolated population in San Bernardino, California (Arbogast et al., 2017). Areas of sympatry with northern flying squirrels have been documented in British Columbia and northern Washington, but to date only Humboldt's flying squirrels have been documented in Oregon and California (Arbogast et al., 2017). Humboldt's flying squirrels are active year‐round throughout their range, with reproduction (breeding to parturition) occurring from April to August (Butler et al., 1991; Driessen et al., 1998; Villa et al., 1999). Parental care is provided exclusively by females, and pups typically wean at about 60 days and sometimes remain longer with their mothers (Booth, 1946; Wells‐Gosling & Heaney, 1984). In populations of Oregon and Washington, pups were found outside the nest from approximately August to October (Villa et al., 1999). Similar to southern and northern flying squirrels, Humboldt's flying squirrel adults are also known to nest communally (Carey et al., 1997). However, there is seasonal and geographic variation in communal nesting behavior, as radiotelemetry studies in Oregon show that communal nesting behavior is more common in winter months compared to summer, while a study in California found only one instance in 2 years of communal nesting (Smith et al., 2011). The seasonal variation is partly driven by reproductive females not nesting with other adults during the summer months (Carey et al., 1997). Thus, the timing of vocalizations, in both nightly and seasonal contexts, may be useful in elucidating the potential behavioral functions of their USVs.

We tested a series of functional hypotheses regarding ultrasonic call‐types in Humboldt's flying squirrels by examining seasonal patterns of their vocalizations in two geographic locations in California. Using ultrasonic recorders, we recorded Humboldt's flying squirrels in the wild in the summer and winter in Humboldt County in northern California and San Bernardino County in southern California. We hypothesized that if call‐types serve different social functions, then call rates of different call‐types might vary seasonally. For example, call‐types produced more often in the summer may be related to breeding activity (Diggins et al., 2020), while call‐types used for anti‐predator functions would be given year‐round. There may also be call‐types used more often in the winter when flying squirrels are more likely to nest communally, which might indicate social cohesion functions. Southern flying squirrels have been observed traveling and foraging together (Madden, 1974; Murrant et al., 2014) and USVs may be used to facilitate social cohesion as squirrels regroup with nestmates when returning to their shared nests (Murrant et al., 2014).

Vocal activity may also vary temporally across flying squirrel nocturnal active period (i.e., dusk to dawn). North American flying squirrels exhibit a biphasic activity pattern, first leaving their nests after sunset and then returning to nests during the night (in some instances multiple times), and then departing again and returning before sunrise for the remainder of the day (Cotton & Parker, 2000; Weigl & Osgood, 1974; Witt, 1992; Wogsland, 2020). Flying squirrels may vocalize more with territorial or mate attraction calls during their first activity period if, similar to hypotheses regarding dawn chorus in birds (Gil & Llusia, 2020), calling is less energetically costly at this time. It is also possible that call‐types given more evenly across nightly active periods may serve foraging functions, and those given more sporadically (no discernable pattern) may be related to reducing the predation risk (i.e., alarm calls).

Nightly vocal activity patterns might also vary seasonally due to balancing thermoregulatory and metabolic needs, and this variation might also be influenced by geographic location. North American flying squirrels have been found to spend a shorter amount of time away from the nest in colder, harsher climatic conditions than in warmer, milder conditions (Cotton & Parker, 2000; Nelson & Sagot, 2018), and in some cases may deviate from a biphasic activity pattern (Cotton & Parker, 2000). Activity pattern variation in Humboldt's flying squirrels in higher elevation San Bernardino County may be more distinct than populations along the northern coast of California in Humboldt County, as San Bernardino has greater temperature shifts. We predicted that Humboldt's flying squirrels would be active for longer periods of time in warmer months of the summer in both counties, but that vocal activity patterns might differ in the winter as Humboldt County has milder temperatures compared to San Bernardino County, which has colder winters.

## 2 MATERIALS AND METHODS

### 2.1 Study areas

We recorded Humboldt's flying squirrels in four areas of Humboldt County in northwestern California and five areas in San Bernardino County in southern California in summer (May–August) and winter (late November–early March) from 2018 to 2022.

#### 2.1.1 Humboldt study areas

The climate of Humboldt County's coast is defined by moderate temperatures that seldom go below freezing in the winter and rarely exceed 25°C during the summer months, averaging between 15 and 20°C with an average annual rainfall of approximately 115 cm (National Oceanic and Atmospheric Administration [NOAA], 2019). We recorded Humboldt's flying squirrels in four areas of Humboldt County known to have the species present based on camera trap studies: Headwaters Forest Reserve (HFR), Arcata Community Forest (ACF), Seawood Cape Preserve (SCP), and Kneeland (KLD). The floral compositions of the HFR, ACF and SCP areas are generally similar, characterized by coast redwoods (*Sequoia sempervirens*) and Douglas firs (*Pseudotsuga menziesii*) (McFarland et al., 2003). HFR contains patches of old‐growth and second‐growth, is near Fortuna, California, USA (407,909.24 m E, 4,497,336.26 m N 10T), and is managed by the Bureau of Land Management and the California Department of Fish and Wildlife. ACF is in Arcata, California, USA (410,922 m E, 4,525,527 m N 10T) and contains a mix of second growth stands which were clear cut 20–30 years ago or thinned ≤10 years (City of Arcata, 2019). SCP is in Trinidad, California, USA (402,863 m E, 4,550,294 N 10T) near the coast of the Pacific Ocean and consists of second growth stands. This preserve is managed by The Wildlands Conservancy, a nonprofit public benefit corporation. Finally, our sites in KLD were on private land in the community of Kneeland, California, USA (423,789 m E, 4,507,053 m N 10T), which are composed of mostly Oregon white oaks (*Quercus garryana*) and Douglas firs (Goff, 2021).

We surveyed seven sites in the HFR area between July 2018 and July 2020 (Table S1). Sites in HFR were in old‐growth patches that were situated approximately 200–500 m in elevation, and sites were from approximately 500 to 1900 m apart. In the ACF area, we surveyed two sites, one in winter 2019 and 2020 and the other in summer 2022. The ACF sites were situated about 640 m apart from each other and situated at ~150–300 m in elevation. We surveyed two sites in the SCP in winter 2021 and in summer 2022. Our SCP sites were located between 70 and 100 m in elevation and roughly 550 m from each other. In KLD, we surveyed two sites, one in winter 2019 and the other in summer 2022. These sites were located between 590 and 870 m in elevation and about 10,000 m apart.

#### 2.1.2 San Bernardino study areas

Study areas in San Bernardino were situated at significantly higher elevations (approximately 1500 to 2500 m above the sea level) than Humboldt County study areas and experience moderately temperate climates with average annual rainfall of approximately 90 cm, annual snowfall averaging approximately 90 cm per year and temperatures ranging from −1°C in winter to 26°C in the summer (NOAA, 2019). Though considered temperate, areas in this montane forest experience more extreme shifts in temperature and climate than areas surveyed in Humboldt. San Bernardino sites were largely characterized by mixed‐conifer forest and dominated by Jeffrey pines (*Pinus jeffreyi*) and white firs (*Abies concolor*), along with areas containing black oaks (*Quercus kelloggii*) and cultivated species at residences (Butler et al., 1991).

We surveyed nine sites in San Bernardino across five study areas based on accessibility, contacting landowners who had previously detected flying squirrels on their properties. We surveyed 3 sites in Lake Arrowhead (LAH) (484,033.1 m E, 3,789,402.9 m N 11S) and two sites in Crestline (CL) (474,953.9 m E, 3,788,053.3 m N 11S), two sites during the winter of 2018 and the summer of 2019, and three sites during the winter and summer of 2020 (Table S1). In the winter of 2019/2020 and summer of 2020, we surveyed 1 site in Big Bear Lake (BBL) (507,047.17 m E, 3,789,132.79 m N 11S), 1 site in Green Valley Lake (GVL) (493,691.63 m E, 3,789,150.45 m N 11S), 1 site in Cedarpines Park (CP) (469,451.14 m E, 3,790,757.24 m N 11S), and 1 site in Smiley Park (SP) (487,595.92 m E, 3,784,308.98 m N 11S). All sites within a study area were greater than 500 m apart (range: 1 to 3 km).

### 2.2 Data collection

We recorded wild flying squirrel calls using Wildlife Acoustics SM4BAT‐FS full‐spectrum ultrasonic recorders (hereafter SM4; Wildlife Acoustics Inc., Maynard, MA, USA). SM4s are designed for acoustic bat surveys and are equally effective at detecting other USV‐producing species. The SM4 recorders were set to a 500 kHz sample rate, at 12 decibels (dB) gain, using a 16 kHz analog high‐pass filter to eliminate unwanted noise signals. Although lower sample rates conserve battery power, higher frequency calls require a higher sample rate to maximize quality (Abrahams, 2018) and we wanted to match settings to previous work done on North American flying squirrels (Diggins et al., 2016). The minimum trigger frequency was set to 12 kHz and the minimum trigger level was set to record within 12 dB of full scale in order to maximize detections. Recordings were set for a minimum duration of 1.5 ms and no maximum duration to record the entirety of phrases (bouts of a single call‐type). Trigger sensitivity was set to high and the minimum time interval was set to 0 to capture a succession of calls in its entirety. Each recorder was scheduled to automatically turn on 1–2 h before sunset and after sunrise each night, and was typically deployed for a duration of 7–14 nights per site, with the exception of some sites being less or more nights depending on when we had access to the sites. The recorder settings were adopted from the SM4 User Guide (Song Meter SM4 FS User Guide 2018, Wildlife Acoustics Inc., Maynard, MA, USA).

We baited all sites using peanut butter suet cakes (Peanut Delight, C&S Products, Iowa, USA) with the SM4 and a Bushnell HD infrared camera (Model # 119836, Bushnell Corporation, Overland Park, Kansas, USA) placed on an adjacent tree roughly 3 m from the bait (similar to Clucas & Atkins, 2022). We used camera trap data to supplement identification of flying squirrel calls by comparing the time of camera detection with the recording timestamp. Cameras were set to take three photos per trigger at 12 M pixels with a 3 s interval at a normal sensor level. The recorder was locked to the trunks of trees and microphones were attached at approximately 2.5 m above the ground and approximately 1.5 m away from the trunk of the tree, facing upwards. To decrease the probability of recording the same individuals across sites in a study area, sites were situated at least 500 m apart during each recording event (Lehmkuhl et al., 2006; Witt, 1992). Methods were in accordance with the United States Animal Welfare Act and were approved by the Cal Poly Humboldt Institutional Animal Care and Use Committee (No. 17/18.W.54‐A and 2021W46‐A).

### 2.3 Call analysis

#### 2.3.1 Identifying call‐types

We sorted call files using SonoBat 4.4.1 (SonoBat, Inc., Arcata, CA, USA). This software was developed to identify bat species and uses a spectrogram and oscillogram for visual representation of each call expressed as frequency (kHz) and time (ms), and uses fast Fourier transform processing at a very high resolution (2048 frequency bins, 128 point buffer, and 013% overlap) to achieve a spectrogram time resolution of 0.025 ms per point. A call represents a single vocalization bordered by an interval of silence (e.g., Gilley et al., 2019). A group of ≥2 calls that are bordered by an interval of silence represents a call‐bout. Call‐bouts were inferred as emanating from a single individual based on the inter‐call durations and consistent amplitude of each call within a call‐bout. We used manual identification by visual inspection of spectrograms in SonoBat using previous descriptions of call‐types to identify calls to call‐types (Eisinger et al., 2016; Gilley, 2013; Gilley et al., 2019; Murrant et al., 2013). Camera trap data validated the presence of flying squirrels at sites where recordings were made, and while misidentification of calls is always possible with manual identification, our extensive work in building a call library for Humboldt's flying squirrels and acoustic analyses of these calls (Farwell et al., 2024), makes this likely negligible for this dataset. For call‐type identification, we viewed calls using a Hann window with a time expansion factor of 10 in SonoBat. We also used the camera trap detections paired with recorder time stamps to verify that flying squirrels were present in the area around the time calls were recorded. Humboldt's flying squirrels are known to use at least 7 distinct ultrasonic call‐types: arc chirps, tonal chirps, downsweeps, upsweeps, trills, arc trills, and three‐toned chirps (Farwell et al., 2024).

#### 2.3.2 Call‐type datasets

We first extracted the timing of calls using Raven Pro 1.4 so that each call had a corresponding time stamp. We then created two vocalization datasets to address nightly and seasonal activity patterns and rates. The first dataset used all call‐types and was created to compare nightly vocal activity patterns between geographic locations and across seasons. This dataset also limited the calls to calling “events”, wherein an event represented independent occurrences of a squirrel vocalizing. This was done to provide general information about when squirrels were vocalizing, but not the rate at which they were producing call‐types. We created events by separating calls by a duration of 30 min, excluding any additional calls within the 30‐min interval. The second dataset focused on the 4 main call‐types we recorded (arc chirps, tonal chirps, upsweeps, and trills) and consisted of all calls recorded of a call‐type for each geographic location to compare the rate of production and the nightly activity patterns between seasons of each call‐type.

### 2.4 Data analyses

#### 2.4.1 Nightly vocal activity patterns

We used the “overlap” package (Version 0.3.4) in R to estimate seasonal nightly vocal activity patterns of flying squirrels (Ridout & Linkie, 2009). We first compared these patterns for all calling events (i.e., all call‐types) within and between geographic locations (Humboldt versus San Bernardino). Then, for each geographic location separately, we compared the nightly patterns for each of the four main call‐types between seasons. The temporal overlap of vocal activity is the overlap coefficient ($\hat{\Delta }$ or D‐hat), which ranges from 0 (no temporal overlap) to 1 (complete temporal overlap). We used the $\hat{\Delta }$_4_ estimator (equation 1) for larger sample sizes (>75) as described in Ridout and Linkie (2009) and Meredith and Ridout (2018):

$${\hat{∆}}_{4}=\frac{1}{2}\left(\frac{1}{n}\sum_{i=1}^{n}\min\left\{1,\frac{\hat{g}\left(x_{i}\right)}{\hat{f}\left(x_{i}\right)}\right\}+\frac{1}{m}\sum_{j=1}^{m}\min\left\{1,\frac{\hat{f}\left(y_{j}\right)}{\hat{g}\left(y_{j}\right)}\right\}\right)$$

where Δ4 describes the coefficient of overlap for populations with sample sizes *n* and *m*, *x* is the timing of vocal activity for the first population over *i* detections, and *y* is the *ij* timing of vocal activity for the second population over *j* detections. To determine vocal activity timing relative to sunrise and sunset, we used the sunTime function in “overlap,” which accounts for the shifting sunrise and sunset times (Nouvellet et al., 2012). To determine if there were significant differences between overlap of variables, we conducted a 2‐sample Anderson‐Darling (AD) test using the R package “kSamples” (Version 1.2–9) with *α* = .05 (Scholz & Zhu, 2019).

#### 2.4.2 Seasonal call‐type rates

We compared call‐type rates between summer and winter for each main call‐type (arc chirps, tonal chirps, upsweeps and trills) for the two geographic locations separately. We determined call‐type rates for each call‐type by quantifying the number of calls per survey night for each site (i.e., dividing the total number of calls by the total number of survey nights). To determine if there were significant differences in call‐type rates between seasons for each call‐type we used non‐parametric tests with exact *p*‐values for determining significance due to our relatively small sample sizes and the data not being normally distributed. For the Humboldt geographic location, where we recorded at different sites in the summer versus winter, we used Wilcoxon rank‐sum tests to test for differences between seasons. For the San Bernardino geographic location, where we sampled the same sites in summer and winter, we used Wilcoxon sign‐rank tests to account for related samples. We calculated effect sizes for all significant results and used the standard of values <0.3 indicating a small effect, 0.3–0.5 a moderate effect, and greater than 0.5 a large effect (Fritz et al., 2012). All statistical tests were run in Program R (Version 2023.12.0 + 369).

## 3 RESULTS

We recorded 583 total calls in Humboldt across 58 nights in the summer (10.1 calls/night) and 1411 calls across 118 nights in the winter (11.9 calls/night), and in San Bernardino we recorded 5637 total calls across 62 nights in the summer (90.9 calls/night) and 3149 calls across 115 nights in the winter (27.3 calls/night). There were two sites where we did not record any flying squirrels on camera or recorder (one each in Humboldt and San Bernardino) so these sites, and an additional site in Humboldt where the recorder was destroyed by a bear, were excluded from the analyses (Table S1).

**FIGURE 1:**
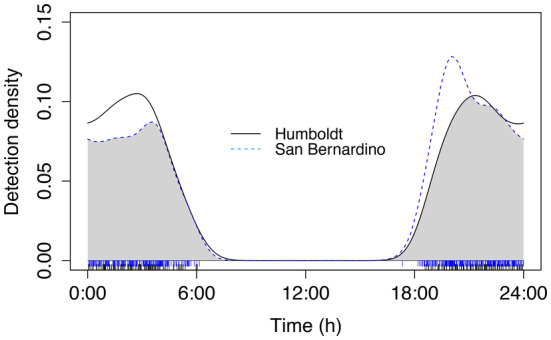
Overlap of vocal activity patterns of all call‐type events of Humboldt's flying squirrels in Humboldt and San Bernardino counties in California. The vertical lines at the bottom of the graph indicate the times of day when calls were recorded (Humboldt County = black lines, San Bernardino County = blue lines).

**FIGURE 2:**
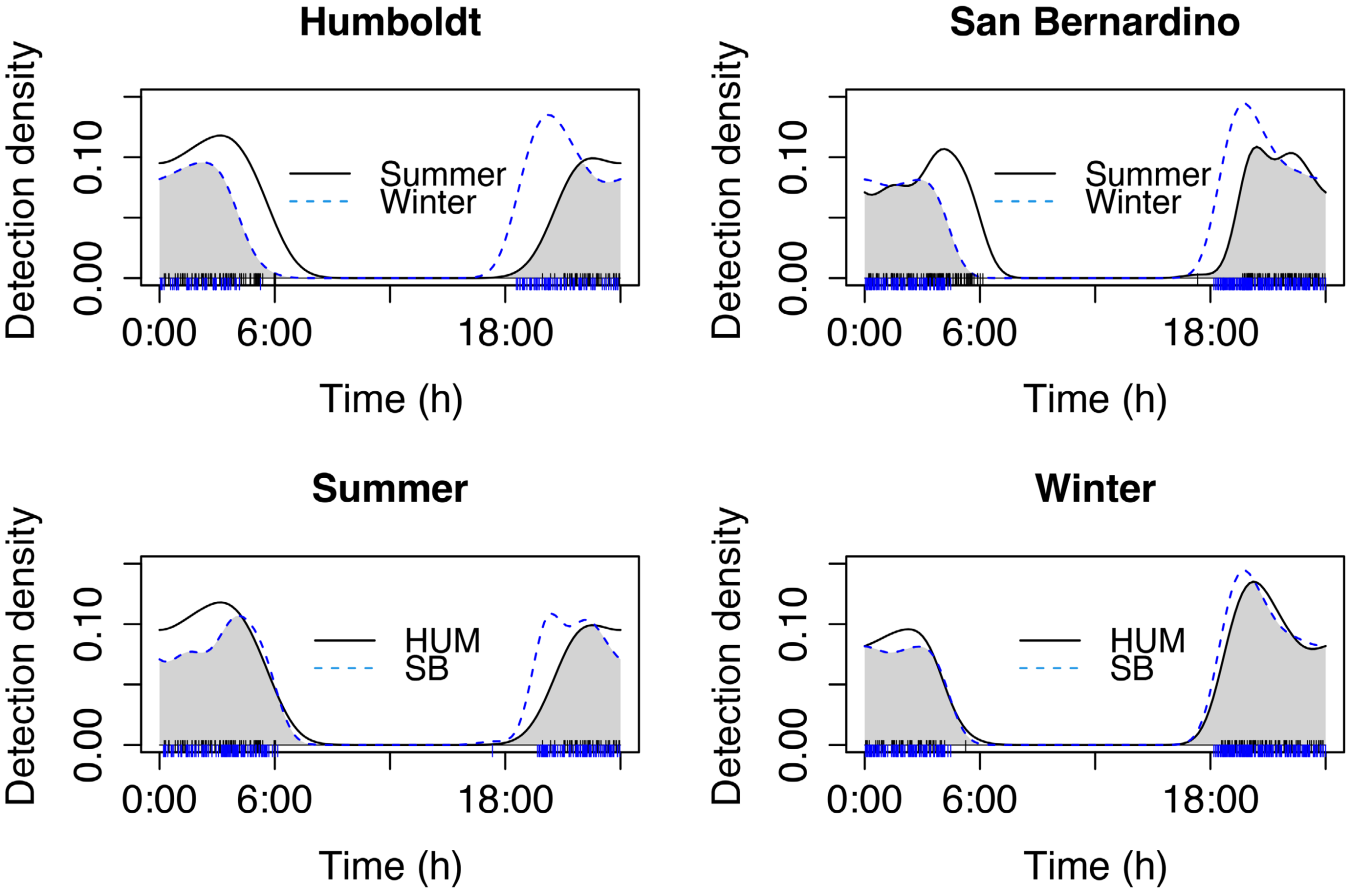
Overlap of vocal activity patterns of all call‐types in summer in Humboldt and San Bernardino counties and winter in Humboldt and San Bernardino counties in California. “HUM” represents Humboldt County and “SB” represents San Bernardino County. The vertical lines at the bottom of the graphs indicate the times of day when calls were recorded (Humboldt County = black lines, San Bernardino County = blue lines).

There were no significant differences in the pattern of activity across the night between counties for all calling events (Figure 1, Table 1), and no differences in pattern of activity across the night between counties across seasons (Figure 2, Table 1). Humboldt's flying squirrel nightly vocal activity patterns tended to display a biphasic pattern with two peaks in activity (Figures 1 and 2).

**TABLE 1:** Overlap estimates (D‐hat) with lower and upper confidence intervals (CI) from bootstrapping for vocal activity of all call‐types of Humboldt's flying squirrels in Humboldt and San Bernardino counties comparing counties and seasons (summer vs. winter). Anderson‐Darling critical value (C. A–D) and test statistic (T. A–D) are also provided.

County	Season	D‐hat	Lower CI	Upper CI	C. A–D	T. A–D	*p*‐value
Humboldt versus San Bernardino	All Seasons	0.913	0.854	0.966	1.288	0.379	.235
Humboldt	Summer versus Winter	0.734	0.632	0.827	1.465	0.616	.184
San Bernardino	Summer versus Winter	0.795	0.727	0.858	1.21	0.271	.265
Humboldt versus San Bernardino	Summer	0.865	0.779	0.939	1.71	0.937	.132
Humboldt versus San Bernardino	Winter	0.943	0.876	0.993	0.269	−0.971	.961

**FIGURE 3:**
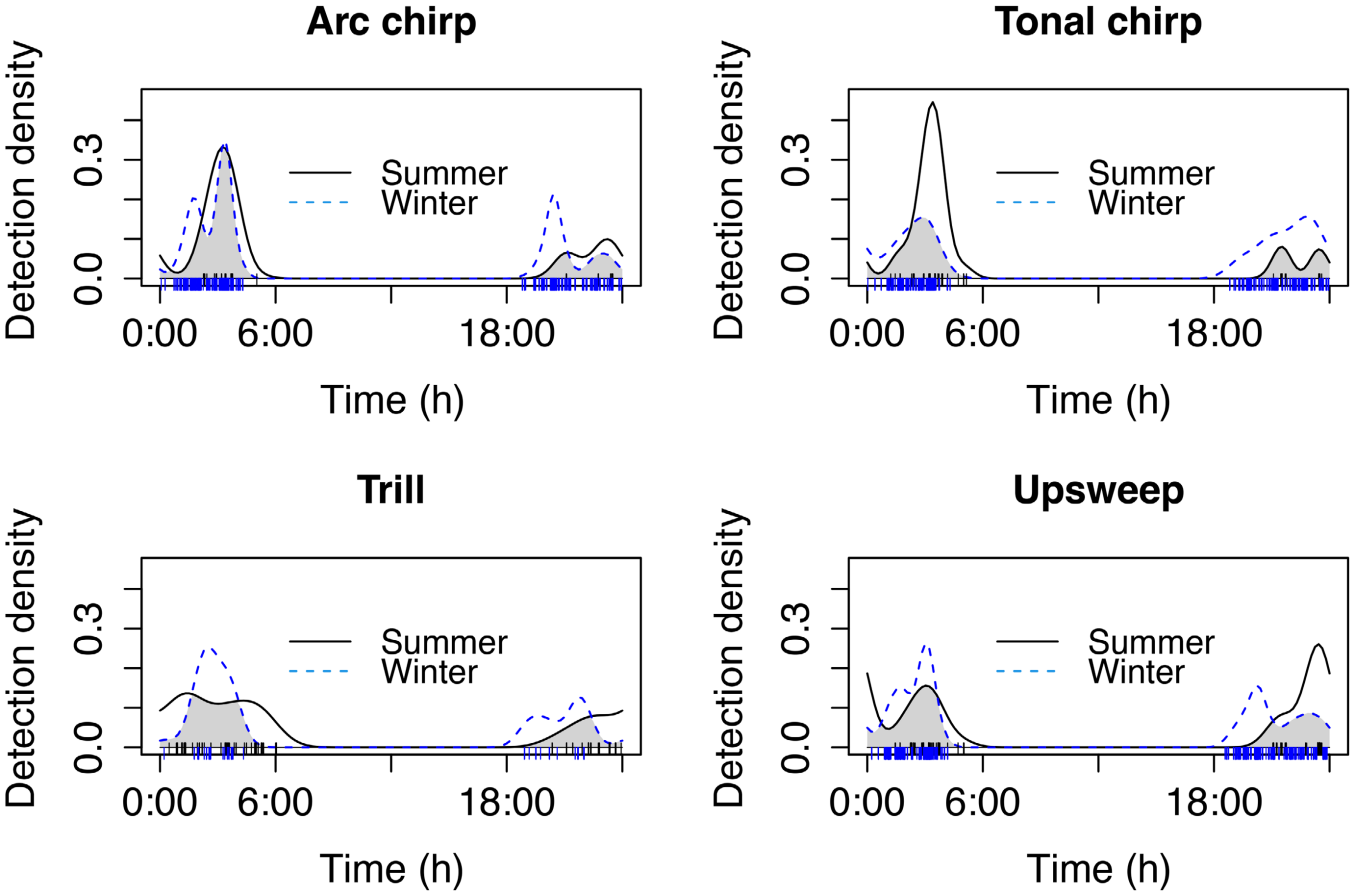
Overlap of temporal patterns of four main call‐types of Humboldt's flying squirrels collected in Humboldt County in California. The vertical lines at the bottom of the graphs indicate the times of day when calls were recorded (Humboldt County = black lines, San Bernardino County = blue lines).

**FIGURE 4:**
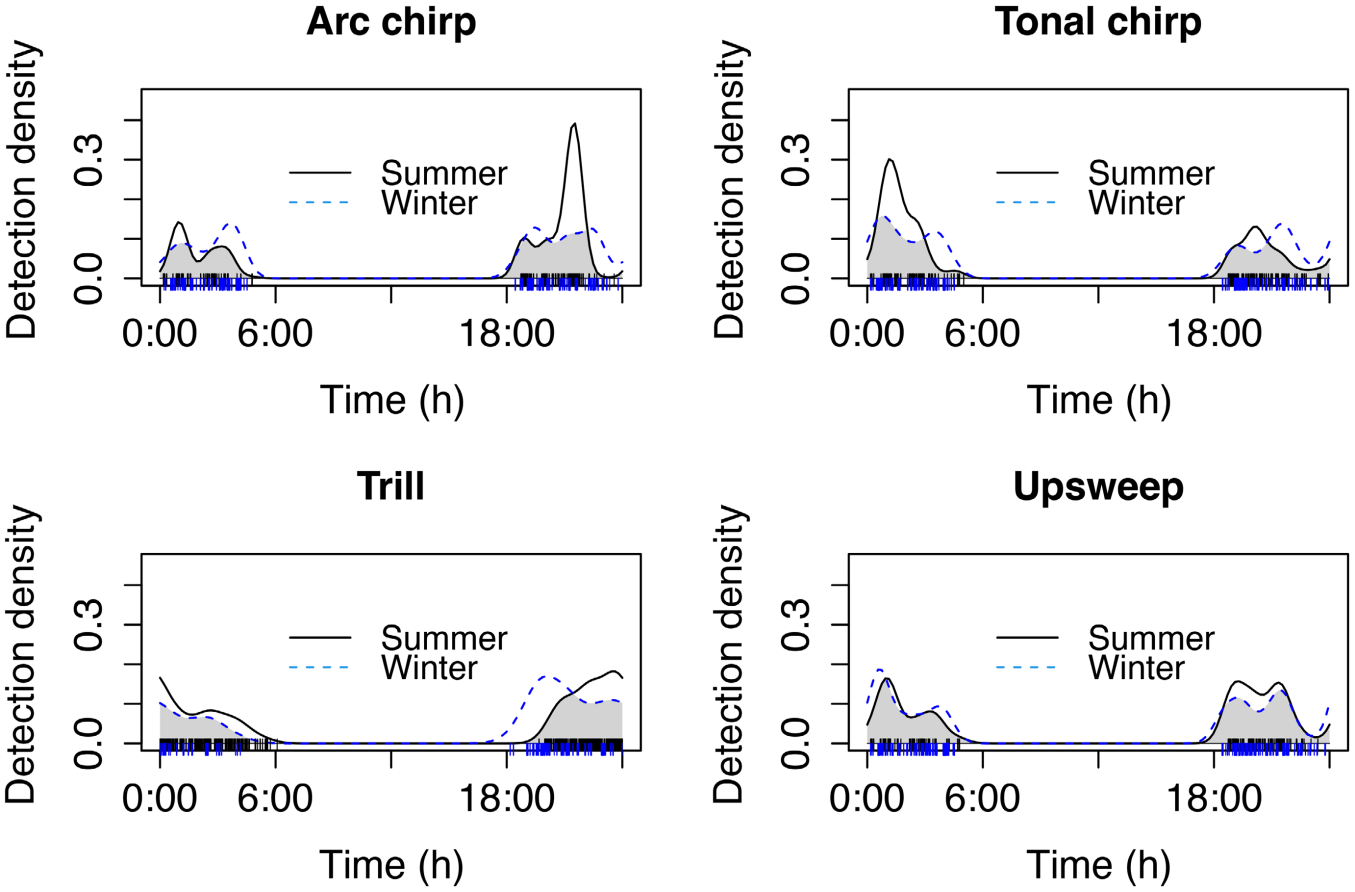
Overlap of temporal patterns of four main call‐types of Humboldt's flying squirrels collected in San Bernardino County in California. The vertical lines at the bottom of the graphs indicate the times of day when calls were recorded (Humboldt County = black lines, San Bernardino County = blue lines).

When comparing the overlap of each of the 4 main call‐types separately, each of the call‐types differed in their nightly pattern between summer and winter in both Humboldt and San Bernardino (Table 2). There was a peak in tonal chirps in the second activity period in summer, but not winter for both counties (Figures 3 and 4). In contrast, the pattern of arc chirps was similar in the second activity period for both counties, but differed in the first activity period, with more in the winter in Humboldt and more in the summer for San Bernardino. In Humboldt, trills peaked in the second activity period, and upsweeps peaked in the first activity period in the summer and in the second activity period in the winter (Figure 3). The patterns of differences for trills and upsweeps were not as clear for San Bernardino and represented more slight shifts in times when these call‐types peaked (Figure 4).

**TABLE 2:** Overlap estimates (D‐hat) with lower and upper confidence intervals (CI) from bootstrapping for vocal activity of each call‐type of Humboldt's flying squirrels in Humboldt and San Bernardino counties comparing summer versus winter. Anderson‐Darling critical value (C. A–D) and test statistic (T. A–D) are also provided.

County	Season/call‐type	D‐hat	Lower CI	Upper CI	C. A–D	T. A–D	*p*‐value
Humboldt	Arc Chirp	0.715	0.660	0.765	6.69	7.47	<.001
Humboldt	Tonal Chirp	0.532	0.468	0.599	21.37	26.77	<.001
Humboldt	Upsweep	0.598	0.553	0.642	103.7	135	<.001
Humboldt	Trill	0.551	0.477	0.625	6.272	6.962	<.001
San Bernardino	Arc Chirp	0.657	0.623	0.689	38.68	49.52	<.001
San Bernardino	Tonal Chirp	0.702	0.668	0.735	35.91	45.88	<.001
San Bernardino	Upsweep	0.868	0.831	0.901	21.62	27.1	<.001
San Bernardino	Trill	0.71	0.652	0.765	13.5	16.42	<.001

There were no statistically significant differences in call rates for arc chirps, tonal chirps, or upsweeps between seasons in Humboldt (Table 3); however, there tended to be a greater rate of trills in summer with a large effect size (*r* = .52), although only marginally significant (*p* = .051). In San Bernardino, all call‐type rates were significantly greater in the summer with large effect sizes (all *r* > .60; Table 4).

**TABLE 3:** Call‐type rate comparison between summer (*n* = 6 sites) and winter (*n* = 8 sites) for Humboldt County.

Call‐type	Median call rate	*Z* score	Effect size	Exact *p*‐value
Arc Chirps	Summer: 0.27 Winter: 1.21	−1.67	NA	.108
Tonal Chirps	Summer: 0.63 Winter: 1.46	−0.97	NA	.362
Upsweeps	Summer: 1.93 Winter: 1.83	−0.06	NA	.982
Trills	Summer: 4.01 Winter: 0.03	1.95	0.52	.051

**TABLE 4:** Call‐type rate comparison between summer and winter (10 pairs of sites) for San Bernardino County.

Call‐type	Median call rate difference (summer‐winter)	*Z* score	Effect size	Exact *p*‐value
Arc Chirps	12.80	2.49	0.79	.001
Tonal Chirps	7.61	2.49	0.79	.001
Upsweeps	7.53	1.98	0.63	.048
Trills	3.48	1.98	0.63	.048

## 4 DISCUSSION

Humboldt's flying squirrels in California displayed a biphasic pattern of vocal activity during their nightly active periods, similar to what has been previously reported for activity patterns using telemetry methods in North American flying squirrel species (Cotton & Parker, 2000; Weigl & Osgood, 1974; Witt, 1992), as well as in several flying squirrel species in Asia (Kuo & Lee, 2012; Suzuki & Ando, 2017) and Eurasia (Airapetyants & Fokin, 2003). Specifically, we found the pattern that Humboldt's flying squirrel vocal activity had two peaks, one after sunset and one before sunrise (Figure 1). When examining the overlap of general nightly vocal activity patterns (i.e., all call‐types together), we found no significant differences in their nightly patterns between summer and winter, or when comparing summer and winter between geographic locations (Humboldt versus San Bernardino). We had predicted that summer vocal activity patterns would be similar between locations; however, we had expected variation in winter patterns between locations due to the differences in winter temperatures. It is possible that flying squirrels in San Bernardino compensate for being active in colder temperatures in similar patterns to those in Humboldt by nesting communally and gaining thermoregulation benefits similar to southern flying squirrels (Murrant et al., 2014). It is also possible that individual squirrels are returning to their nest sites multiple times over the night as found in Humboldt's flying squirrels in Oregon and Washington (Carey et al., 1997), but this is not reflected in the overall activity pattern of all squirrels.

When examining the nightly vocal activity patterns of the main call‐types in Humboldt's flying squirrel separately, there was variation between seasons, with some call‐type production peaking in the first active period of the night (arc chirps in the summer in Humboldt and in the winter in San Bernardino), while others peaked in the second active period (tonal chirps in both locations in the summer). This variation in timing might be due to the differences in behavioral requirements in the summer compared to the winter, for example if communal nesting occurs at higher rates in the winter and vocalizations are needed for social cohesion (Murrant et al., 2014). If arc chirps or tonal chirps are potential alarm calls, they might vary in their nightly and seasonal patterns due to variation in predation risk (Abbey‐Lee et al., 2016). Perceived predation risk might also influence production of vocalizations following the predator eavesdropping avoidance hypothesis (Dabelsteen, 2005) where prey refrain from or reduce calling to avoid being detected and located by predators. For example, veeries (*Catharus fuscescens*), a small songbird, decreased their territorial vocalizations at dusk when barred owl (*Strix varia*) predation risk was simulated (Schmidt & Belinsky, 2013). Both Humboldt and San Bernardino populations are prey of spotted owl species (*Strix occidentalis*; Smith et al., 1999; Wilkinson et al., 2023; Zabel et al., 1995); however, spotted owls would likely not be able to hear flying squirrel USVs, as owl species that have been tested cannot hear in the ultrasonic (reviewed in Dyson et al., 1998). In contrast, it is possible that carnivore predators of flying squirrels could hear flying squirrel USVs (Arch & Narins, 2008; Heffner & Heffner, 1985). Experimental simulation of predation risk could provide insight into if and what types of calls flying squirrels use when predation risk is increased.

Call rates of Humboldt's flying squirrel vocalizations did not differ significantly between summer and winter for arc chirps, tonal chirps, and upsweeps in Humboldt, but these rates were all higher in the summer in San Bernardino. The difference between geographic locations would need to be explored further, but flying squirrels in Humboldt tended to have lower call rates overall compared to San Bernardino, so differences between seasons may be more difficult to detect. The trill call‐type was produced at higher rates in the summer than in the winter in San Bernardino, and the pattern was similar, although only marginally significant, in Humboldt. Similarly, Northern flying squirrels were found to increase the number of trills in late spring in North Carolina and researchers hypothesized that trills may be a breeding call (Diggins et al., 2020). Trills being produced at higher rates in the summer compared to winter by Humboldt's flying squirrels in California may be related to breeding behavior that occurs from April to August. In addition, territorial behavior has been shown to increase during the breeding season in North American flying squirrel species, in particular in females (Jacques et al., 2017; Madden, 1974; Smith et al., 2011). Female flying squirrels have been found to not nest communally when rearing their young in the summer and display less home range overlap than males.

Taken together, the nightly patterns of the different call‐types and their rates between seasons provide only limited insight into the function of the call‐types. Perhaps the most notable difference is the greater call rate of trills in the summer in both geographic locations, which suggests a breeding and/or territorial function. Trills are also the most acoustically complex calls given by Humboldt's flying squirrels (Farwell et al., 2024), so it is also possible that different forms of trills might serve different or multiple functions. For instance, Gilley et al. (2019) found that juvenile southern flying squirrels recorded in captivity gave an acoustically distinct juvenile trill call and researchers have hypothesized that trills may be a juvenile separation call (Diggins et al., 2020). These possible multiple functions of the trill call‐type warrant further study. The simpler acoustic structures of the chirp‐like calls may suggest they could be alarm or contact calls, but further investigation would also be needed. In addition to the four main call‐types we recorded at high numbers, there were three other call‐types that were produced at much lower rates ‐ downsweeps, arc‐trills and three‐toned chirps ‐ and in one case only in San Bernardino (three‐toned chirps; see Farwell et al., 2024). The low number of these call‐types precluded temporal and production rate analyses across seasons, but their low number in general might indicate they play a more specific function (e.g., communication between females and their pups), or that they might be calls that are only given by young flying squirrels (Diggins, 2021). Overall, a finer scale analyses that examined call‐type rates across all months of the year might elucidate changes of patterns and call rates over the year and provide further insight into the function of the call‐types. Moreover, behavioral studies that additionally capture the context in which calls are given might also be possible with experimental approaches in the wild with both recorders and camera traps (Buxton et al., 2018).

Our study has several limitations in part due to the challenges of working with free‐living animals and recording them remotely. First, we could not identify individuals or their age or sex from the recordings, nor, in some cases, could we know if there was more than one squirrel calling at a site. Although we had a camera trap at each site to confirm the presence of flying squirrels at the site, we could not reliably estimate the number of squirrels because we could not know if every vocalizing squirrel was also caught on camera, or if it was the same squirrel coming and going, or more than one squirrel. Second, to increase the probability of recording calls and to get photo confirmation of flying squirrel presence, we baited our camera and ultrasonic recorder sites. This bait might have increased social interactions that might not have occurred otherwise, and this might have influenced the production of certain call‐types; however, any influence would have been similar in the summer and winter. Future studies could try not using bait by increasing the number of recorder stations to increase the probability of recording calls, and radio‐telemetry/GPS collars or passive integrated transponders (PIT) could be used to know where individuals are located in relation to recorders (e.g., Garroway et al., 2013).

Examining the nightly and seasonal patterns of vocalizations of the Humboldt's flying squirrel represents a first step in investigating the function of their multiple call‐types. Of squirrel species, flying squirrel vocalizations remain the least studied and little is known about their potential functions (Diggins, 2021). Understanding the activity patterns of flying squirrels in general is also important when considering conservation of these species. As the thermoregulatory needs of smaller mammals like flying squirrels are higher than of larger animals (Riek & Geiser, 2013), shifts in temperature and climate could have a significant influence on the activity patterns of flying squirrels. Establishing a baseline of the activity patterns of flying squirrels is fundamental for understanding the degree to which climate change (Schweiger & Frey, 2021) and human disturbance (Clark et al., 2021) may affect their behavior and long‐term survival. Using ultrasonic recorders to monitor flying squirrels provides a remote and non‐invasive method to determine their presence and activity patterns in the wild (Diggins et al., 2016). Understanding the function of call‐types produced by flying squirrels could aid in determining age and breeding status from the call‐types recorded, or these demographic characteristics could be determined by acoustic analysis of the structure of certain call‐types (e.g., Owings et al., 1986). These possibilities could increase the type of information that could be collected during acoustic surveys of flying squirrels and could provide an alternative to live trapping to obtain demographic information.

## AUTHOR CONTRIBUTIONS

**Travis A. Farwell:** Conceptualization (equal); data curation (equal); formal analysis (equal); investigation (equal); methodology (equal); visualization (equal); writing – original draft (equal); writing – review and editing (equal). **Barbara Clucas:** Conceptualization (equal); data curation (equal); formal analysis (equal); funding acquisition (lead); investigation (equal); methodology (equal); visualization (equal); writing – original draft (equal); writing – review and editing (equal).

## CONFLICT OF INTEREST STATEMENT

The authors declare they have no conflicts of interest.

## Supporting information


Table S1.

## Data Availability

Data are available in the supporting information document.
