# Supplementary material for: Seasonal variation in the ultrasonic vocal activity of Humboldt's flying squirrel (Glaucomys oregonensis)
Source: Ecol Evol. 2024 Sep 23;14(9):e70344. doi: 10.1002/ece3.70344 (PMC11419791; doi:10.1002/ece3.70344)
Supplement: Supplementary file 1 — Table S1. [file ECE3-14-e70344-s001.docx]

**Supporting Information**

**Seasonal variation in ultrasonic vocal activity of Humboldt’s flying squirrel (*Glaucomys oregonensis*)**

Travis A. Farwell^1^ and Barbara Clucas^1^

*^1^Department of Wildlife, Cal Poly Humboldt, Arcata, California 95521*

**Corresponding author: Barbara Clucas, Department of Wildlife, Cal Poly Humboldt, 1 Harpst St, Arcata, CA 95521,* [*barbara.clucas@humboldt.edu*](mailto:barbara.clucas@humboldt.edu)*, 707-826-5651*

Table S1. Seasonal call-type data of four main call-types of Humboldt’s flying squirrels in Humboldt and San Bernardino counties in summer and winter.

| **County/**  **Season** | **Month(s)** | **Year(s)** | **Area** | Site Number | Number Nights | Arc Chirp | Tonal Chirp | Upsweep | Trill | Total |
| --- | --- | --- | --- | --- | --- | --- | --- | --- | --- | --- |
| Humboldt Summer | June | 2022 | ACF | 5 | 13 | 5 | 88 | 42 | 70 | 205 |
| Summer | July | 2018 | HFR | 18 | 13 | 31 | 9 | 69 | 60 | 169 |
| Summer | July-August | 2019 | HFR | 21 | 19 | 4 | 11 | 30 | 10 | 55 |
| Summer | July-August | 2019 | HFR | 23 | 19 | 1 | 21 | 15 | 8 | 45 |
| Summer | July | 2020 | HFR | 25 | 3 | 1 | 0 | 0 | 11 | 12 |
| Summer | July | 2022 | KLD | 2 | 14 | 0 | 4 | 32 | 61 | 97 |
| Summer | August | 2022 | SCP† | 1 | 23 | 0 | 0 | 0 | 0 | 0 |
| Summer | August | 2022 | SCP‡ | 5 | NA | - | - | - | - | - |
| HumboldtWinter | February | 2019 | ACF | 9 | 14 | 206 | 47 | 153 | 0 | 406 |
| Winter | March | 2020 | ACF | 9 | 16 | 106 | 12 | 20 | 0 | 138 |
| Winter | November | 2018 | HFR | 7 | 14 | 2 | 18 | 0 | 35 | 55 |
| Winter | March | 2019 | HFR | 14 | 11 | 6 | 6 | 3 | 57 | 72 |
| Winter | January | 2019 | HFR | 20 | 14 | 7 | 23 | 36 | 0 | 66 |
| Winter | February-March | 2019 | KLD | 1 | 15 | 1 | 0 | 6 | 10 | 17 |
| Winter | December-January | 2021 | SCP | 1 | 17 | 136 | 103 | 231 | 1 | 471 |
| Winter | December-January | 2021 | SCP | 5 | 17 | 32 | 112 | 41 | 0 | 185 |
| San Bernardino Summer | May and June | 2020 | BBL | 1 | 4 | 456 | 426 | 210 | 77 | 1169 |
| Summer | June | 2019 | CL | 1 | 9 | 0 | 11 | 5 | 1145 | 1161 |
| Summer | June | 2020 | CL | 1 | 1 | 20 | 35 | 22 | 13 | 90 |
| Summer | June | 2020 | CL | 2 | 2 | 17 | 28 | 15 | 3 | 63 |
| Summer | June | 2020 | CP**§** | 1 | 7 | 1 | 0 | 0 | 62 | 63 |
| Summer | May-June | 2020 | GVL | 1 | 8 | 179 | 115 | 110 | 48 | 452 |
| Summer | June | 2019 | LA | 1 | 3 | 406 | 440 | 167 | 24 | 1037 |
| Summer | June | 2020 | LA | 1 | 6 | 645 | 47 | 70 | 17 | 779 |
| Summer | May-June | 2020 | LA | 2 | 8 | 22 | 23 | 91 | 24 | 160 |
| Summer | May-June | 2020 | LA | 3 | 5 | 36 | 51 | 37 | 83 | 207 |
| Summer | June | 2020 | SP | 1 | 9 | 157 | 23 | 54 | 4 | 238 |
| San Bernardino Winter | December | 2019 | BBL | 1 | 14 | 104 | 117 | 243 | 17 | 481 |
| Winter | December | 2018 | CL | 1 | 6 | 18 | 41 | 57 | 0 | 116 |
| Winter | January | 2020 | CL | 1 | 13 | 107 | 124 | 118 | 112 | 461 |
| Winter | January | 2020 | CL | 2 | 4 | 32 | 20 | 33 | 19 | 104 |
| Winter | January | 2020 | CP**†** | 1 | 14 | 0 | 0 | 0 | 0 | 0 |
| Winter | January | 2020 | GVL | 1 | 13 | 111 | 60 | 59 | 58 | 288 |
| Winter | December | 2018 | LA | 1 | 14 | 191 | 401 | 707 | 5 | 1304 |
| Winter | January | 2020 | LA | 1 | 13 | 38 | 21 | 26 | 15 | 100 |
| Winter | December | 2019 | LA | 2 | 14 | 6 | 8 | 12 | 6 | 32 |
| Winter | December | 2019 | LA | 3 | 11 | 17 | 44 | 87 | 12 | 160 |
| Winter | January | 2020 | SP | 1 | 13 | 25 | 8 | 2 | 33 | 68 |

**†**No flying squirrels recorded at site on ultrasonic recorder or camera, not used in any analysis

**‡** Bear destroyed recorder and camera the night the station set up, not used in any analyses

**§**No flying squirrels recorded at site in corresponding winter surveys, not used in analysis of call rates
